# Pooled dataset on entrepreneurial characteristics of undergraduates in selected universities in Nigeria

**DOI:** 10.1016/j.dib.2021.107718

**Published:** 2021-12-16

**Authors:** Adedayo Olofinyehun, Abiodun Egbetokun, Caleb Adelowo

**Affiliations:** a National Centre for Technology Management, Ile-Ife, Nigeria; b North-West University, Potchefstroom, South Africa

**Keywords:** Entrepreneurship interest, Entrepreneurship experience, Undergraduates, Nigeria, dataset, ABU, Ahmadu-Belllo University, CEPR, Centre for Economic Policy Research, CGPA, Cumulative Grade Point Average, CU, Covenant University, DFID, Department for International Development, DiD, Difference-in-Differences, EDC, Entrepreneurship Development Centre, FMSTI, Federal Ministry of Science, Technology and Innovation, FUNAAB, Federal University of Agriculture, Abeokuta, FUTMinna, Federal University of Technology, Minna, GEM, Global Entrepreneurship Monitor, GUESSS, Global University Entrepreneurial Spirit Students’ Survey, JAMB, Joint Admissions and Matriculations Board, NACETEM, National Centre for Technology Management, NUC, National Universities Commission, OAU, Obafemi Awolowo University, PECS, Personal Entrepreneurship Characteristics, PEDL, Private Enterprise Development in Low-Income Countries, STEP-B, Science and Technology Education Post-Basic, UNILAG, University of Lagos

## Abstract

This pooled dataset presents data collected through four (4) sequential cross-sectional surveys of undergraduates in six (6) selected Nigerian universities. The data were collected from a total of 12,615 undergraduates studying courses in the social sciences, sciences and engineering disciplines. The surveys assessed entrepreneurship interest, background and experience of the respondents. The dataset is useful for research, policy and practice in several ways. Coming from surveys repeated at intervals of between four and five years, the dataset allows for an assessment of the impacts of the compulsory entrepreneurship training that was introduced in the Nigerian university system at about the time of the first survey. It can also be used to quantify the potential pool of future entrepreneurs among the highly educated Nigerian youth. Additionally, the dataset presents a full entrepreneurship profile of a very large pooled cross-sectional sample of educated young people in the largest and most populous nation in sub-Saharan Africa. Consequently, researchers, policymakers, donors and other development practitioners seeking to characterize and design appropriate interventions for youths in the developing world will find this dataset valuable.

**Specification Table**

**Table:** 

Subject	Entrepreneurship
Specific subject area	Entrepreneurship
Type of data	Primary data Table
How data were acquired	Data were collected using structured questionnaires administered to university undergraduates on campuses. The questionnaires included both close-ended and Likert-scale questions. A representative version of the questionnaires is provided as a supplementary file.
Data format	Raw
Parameters for data collection	The data were collected through repeated cross-sectional surveys from a total of 12,615 university undergraduates who were studying social science, science or engineering courses and between the second and fifth years of study.
Description of data collection	The data collection was through repeated cross-sectional surveys in six universities. The surveys took place in 2007, 2010/11, 2016 and 2020/21. The selection of the universities was based on their status as at 2011 regarding implementation of a compulsory entrepreneurship training policy introduced in 2006. In each of the selected universities, a random sample of social science, science and engineering undergraduates was taken.
Data source location	Institution: National Centre for Technology Management City/Town/Region: Ile-Ife, Osun State Country: Nigeria
Data accessibility	In a public repository Repository name: Mendeley Data Data identification number: Mendeley Data, V2, doi: 10.17632/zgkxb5tvpg.2 Direct URL to data: https://data.mendeley.com/datasets/zgkxb5tvpg/2
Related research article	A.O. Olofinyehun, C.M. Adelowo, A.A. Egbetokun, The supply of high-quality entrepreneurs in developing countries: evidence from Nigeria, Science and Public Policy 45(2) 269-282. https://doi.org/10.1093/scipol/scx065

**Value of the Data**

• The dataset is useful for quantifying and characterizing potential educated entrepreneurs in Nigeria and for understanding the correlates of their motivation and interest.
• The timing of the surveys that generated the pooled dataset allows a near perfect quasi-experimental design for assessing the impacts on students of the initiative of the National Universities Commission (NUC) that mandated every university in Nigeria to introduce compulsory entrepreneurship courses. The first survey, collected at about the time of the introduction of the policy, would be a good baseline. The second survey was collected when only half of the included universities had implemented the policy. The second and the third surveys were collected when all the universities had implemented the policy, representing post-intervention follow-ups.
• Researchers in the field of entrepreneurship and development studies can use the data to compare with other countries, determine further research and support systematic reviews in the future.
• Governments and donors can especially find the data useful in designing strategies and programmes to support entrepreneurship development among educated young people in developing countries. Nigeria is a typical developing country in terms of its size and economy; lessons from Nigeria can be usefully adaptable for the rest of the developing world.

## 1 Data Description

The pooled dataset is from a set of four repeated cross-sectional surveys of undergraduates in six large Nigerian universities. The respondents are undergraduates studying engineering, science and social science courses in their second year upward; first year students were not covered because of the possibility of them not yet being affected by the compulsory entrepreneurship policy.

All surveys were self-administered. Survey instruments were hand-delivered to the respondents and later retrieved by a trained enumerator who was also on hand to provide any necessary clarifications. The survey instrument changed a bit over time, but the changes are minor. Specifically, due to the variations in the primary focus of each of the surveys, some questions were phrased differently across the surveys, but the core items remained the same throughout. The latest and most representative of the survey instruments is included as a supplementary file in the data repository mentioned in the specification table above.

In addition to background information of the respondents including age, gender, marital status, religion, ethnic origin, course and level of study, current cumulative grade point average (CGPA), all four surveys included the following sections: parental entrepreneurial experience/background; entrepreneurial experience, motivation and interest; entrepreneurship training; university entrepreneurial context and potential. The last two surveys also contain a comprehensive assessment of personal entrepreneurial characteristics (PECs). Structured questionnaires were used to collect information from the respondents. The questionnaires included both close-ended and Likert-scale questions with some open-ended questions that sought to elicit detailed explanations, as necessary.

The variables captured include the following:

(i) Personal information (biodata);This section contains questions on respondents’ personal information including age as at last birthday, sex, marital status, religion and ethnic origin;
(ii) Educational information;This section contains questions on respondents’ course and present level of study, as well as present CGPA;
(iii) Family background;Here we sought to profile the respondents in terms of their parents’ highest education qualifications, whether or not they have run a business of their own before, what kind of business, and whether or not the business is still in operation;
(iv) Entrepreneurship/business experience;This section asks questions on whether or not the respondents have run a business before, what kind of business it was; their motivation for running the business; whether they were the initiator or a partner in the business; as well as the reason for never running a business, for those who never had a business of their own;
(v) Entrepreneurial attitude;This section contains questions on respondents’ interest in starting a business of their own; how strong the interest was; and whether or not they had a written business plan;
(vi) Entrepreneurship education;Here we sought to find out if the respondents have taken entrepreneurship courses before, where, when and what type of course it was; as well as what they gained from the course;
(vii) University context and entrepreneurial potential;This section seeks the respondents’ views on how their school environment supports entrepreneurship activities;
(viii) Personal entrepreneurship characteristics (PECS);This section is a group of 55 5-point likert-scale standard assessments questions that can be used to measure/characterize individuals’ entrepreneurship characteristics.

## 2 Profile of Respondents

The profile of the respondents is presented in Table 1. As could be expected, almost all (95%) of them are between age 16 and 30 years, half of them are between 21 and 25 years, and over 90% of them are single. There are more males (61.3%) than females in the dataset. This is, however, not a reflection of the sex distribution of undergraduates, either in Nigeria generally or in the selected schools, as other studies/surveys have shown a near equal distribution. The respondents are evenly distributed across the three major disciplines – engineering, sciences and social science-based courses – though most are from sciences. In terms of ethnic origin, the three major tribes – Hausa, Igbo and Yoruba – are well represented; and all additional tribes (including, for instance, Efik, Tiv, Ijaw, etc) are bundled under the ‘Others’ category. The distribution of respondents by present level of study and present CGPA are also shown in Table 1.

**Table 1:** Profile of respondents.

Attribute	No.	%
*Age*		
< 16 years	34	0.3
16–20 years	4162	33
21–25 years	6374	50.5
26–30 years	1458	11.6
31–35 years	80	0.6
36– 40 years	44	0.3
Above 40 years	26	0.2
No response	437	3.5

*Marital status*		

Single	11,662	92.4
Married	614	4.9
Divorced/separated/widowed	61	0.5
No response	278	2.2

*Sex*		

Male	7732	61.3
Female	4753	37.7
No response	130	1

*Discipline of study*		

Engineering-based	3711	29.4
Science-based	4932	39.1
Social science-based	3136	24.9
Others	639	5.1
No response	194	1.5

*Ethnic origin*		

Hausa	1779	14.1
Igbo	1867	14.8
Yoruba	6427	50.9
Others	1887	15
No response	655	5.2

*Present level of study*		

500 level	2645	21
400 level	3303	26.2
300 level	63,832	30.3
200 level	2571	20.4
No response	273	2.2

*Present CGPA*		

< 1.00	14	0.1
1.00+	153	1.2
2.00+	1491	11.8
3.00+	4243	33.6
4.00+	3352	26.6
5	1037	8.2
No response	2325	18.4

## 3 Experimental Design, Materials and Methods

*(a) Background*

In 2006, the Nigerian government sanctioned a public policy intervention mandating all universities in Nigeria to introduce compulsory entrepreneurship courses across all disciplines and to establish entrepreneurship development centres (EDCs). The NUC designed the policy and monitored compliance among universities. In most universities, the compulsory entrepreneurship course is essentially a compulsory elective which undergraduates have to take somewhere between their second and final years of study. The final year is the fourth or fifth year of study depending on the course of study. Thus, once an individual is registered in a university where the policy is already in force, he is bound to complete the compulsory entrepreneurship course before the end of his/her regular course programme. Implementation of the policy began around 2007 but took effect at different times within different universities up until around 2013.

The dataset we present is pooled from four separate cross-sectional surveys. The first two surveys were designed to capture the entrepreneurship characteristics of Nigerian undergraduates, while the last two were designed for an impact evaluation study, which aimed to assess the impact of the policy on entrepreneurial interest and practice of undergraduates. The first survey was collected between November 2006 and February 2007 from twenty-five tertiary institutions in Nigeria. The second and larger survey was collected between 2010 and 2011 from fifty-five tertiary institutions. In 2016, a quasi- experimental study assessing the impact of the NUC policy, Olofinyehun et al. [3] utilized the pooled dataset from 2007-11. The evaluation study identified six of the largest and historically best performing^1^ universities^2^ in the dataset to set up a difference-in-difference (DiD) model. The model requires that some of the universities must have complied with the NUC policy (treated sample) and some must not have done so (control sample).

The six universities that were selected included three that had introduced the compulsory entrepreneurship course before 2007 and another three that had introduced it only by 2011. To extend the evaluation analysis, two other surveys – one in May/June 2016 and another between 2020 and 2021 – were then conducted in those six universities only. For the final dataset that we describe in this paper, we extracted data for the six universities from the first two surveys and merged it with the last two surveys.

*(b) Sampling*

For the first two surveys, respondents were selected by a rigorous systematic random sampling procedure. Table 2 provides details on the distribution of sampled institutions by type. The first survey included twenty-five (at that time, about a fifth of all officially registered) tertiary institutions while the second included fifty-five (at that time, over a third of all officially registered) tertiary institutions. The sample of tertiary institutions for the two surveys was drawn from the list of schools in the latest examination brochures published by the Joint Admissions and Matriculations Board (JAMB) as at the time of commencement of each of the surveys. This sampling frame is intrinsically reliable since JAMB is the principal authority responsible for conducting admission examinations into all categories of institutions covered by the study and it is known that JAMB's institutional listings include only accredited institutions and courses.

**Table 2:** Distribution of sampled institutions for the first two surveys.

	2007				2010/11		
Institution type	Total	Sample	Total %		Total	Sample	Total %
University	65	13	20		92	31	33.7
Polytechnic	51	9	17.6		52	17	32.7
College of Education (Technical)	8	3	37.5		7	7	100
Total	124	25	20.2		151	55	36.4

The population of tertiary institutions in Nigeria was clustered respectively by location, age, ownership, and availability of several courses within the three broad disciplines focused in the surveys (science, social science, and engineering). For instance, all universities located in South-Western Nigeria will belong to the same location cluster but different age, ownership, and discipline clusters. The final sample of schools was randomly selected across clusters. Respectively, the population of science, social science, and engineering undergraduates in the selected tertiary institutions was clustered by their specific course and current year of study. For example, all mechanical engineering undergraduates will belong to the same course cluster but different year-of-study clusters. First-year students were excluded since extant entrepreneurship-related policies and programmes in their respective schools will only have minimally influenced them, if it does at all. The final sample of respondents was randomly selected across clusters. To make the final sample as representative as possible, an equal number of respondents was targeted from each specific cluster across the institutions. A census was taken wherever the cluster size was not more than twenty. The last two smaller and more specific surveys were purposive by selection of universities, but sampling within the universities followed the same random selection as in the first two larger surveys. Table 3 presents the breakdown of the pooled dataset across the surveys and the six selected universities. As already hinted, the present dataset was constructed by merging data from the third and last survey with those from the first and second surveys. Ultimately, the pooled cross-sectional dataset includes six universities, which are Ahmadu-Bello University (ABU); Covenant University (CU); Federal University of Agriculture, Abeokuta (FUNAAB); Federal University of Technology, Minna (FUTMinna); Obafemi Awolowo University (OAU); and University of Lagos (UNILAG).

**Table 3:** Breakdown of the dataset by institution and year of survey.

Year of survey Institutions	2007	2010/11	2016	2020/21	Total
Ahmadu Bello University (ABU)	357	964	591	649	**2,561**
Covenant University (CU)	281	344	534	744	**1,903**
Federal University of Agriculture, Abeokuta (FUNAAB)	301	17*	562	521	**1,401**
Federal University of Technology, Minna (FUTMinna)	79	954	500	623	**2,156**
Obafemi Awolowo University, Ile-Ife (OAU)	360	641	583	646	**2,230**
University of Lagos, Akoka (UNILAG)	371	821	507	665	**2,364**
Total	**1,749**	**3,741**	**3,277**	**3,848**	**12,615**

⁎ FUNAAB was probably on holiday at the time of the survey as university calendar is not uniform in Nigeria, or most of the data for FUNAAB in the survey was cleaned off by the impact evaluation regression analysis

*(c) Questionnaire development and validation*

The survey and invariably the study instrument was premised on the theory of planned behaviour as advanced by Ajzen [1,2]. The theory postulates that intention to perform an act is influenced by a number of factors including attitude towards a behaviour, subjective norm and perceived behavioural control. Therefore, willingness to start a business (entrepreneurship) is contingent on a number of factors including personality traits, motivation and entrepreneurship ecosystem.

The questionnaire used in this study is a result of adapting several variables used in previous studies, mainly, variables drawn from Wang and Wong [4], the Global Entrepreneurship Monitor (GEM)^3^ and the Global University Entrepreneurial Spirit Students’ Survey (GUESSS)^4^. The PEC items were adapted from a study conducted by Management Systems International in 1985^5^. Given that we were starting out from widely validated instruments, re-inventing the wheel of scale development was not particularly necessary. However, it was still necessary to ensure contextual relevance of the measures. Thus, brainstorming sessions were held to systematically identify the relevant variables and discuss the most suitable question wordings. To maximize data quality and to ensure that the questionnaire was not burdensome to respondents, the number of pages in the questionnaire was kept as few as possible.

The final questionnaire that emerged from the brainstorming sessions was then validated through a pilot survey. The pilot survey was carried out in a university (the University of Ibadan) and a polytechnic (The Polytechnic, Ibadan) in the South-Western region of Nigeria. These two institutions are among the oldest, largest and most diverse in terms of ethnic representation in the country and the randomly selected pilot sample was about 200 undergraduates from each institution. Feedback from the pilot survey indicated that the items in the questionnaire gathered the expected information except for a few minor issues such as a misunderstanding of grades due to differences between universities and polytechnics. Such issues were rectified in the final survey instrument. As mentioned earlier, the subsequent surveys adapted the original questionnaire.

## Ethics Statement

In implementing the surveys, informed consent was a key ethical issue that was considered. Every participant gave their consent before questionnaires were administered. Essentially, they were informed about what participation in the study would entail. Every questionnaire was prefaced by information that explained the purpose of the study and the role of the implementing agency/researchers.

## Funding

Each of the four surveys from which the pooled dataset was generated were funded by different bodies, viz:

(i) 2007 survey was funded by the Federal government of Nigeria through the FMSTI;
(ii) 2010/11 survey was funded by the World Bank through the Science and Technology Education Post-Basic (STEP-B) Programme;
(iii) 2016 survey was funded by the Private Enterprise Development in Low-Income Countries (PEDL) Initiative, a joint initiative of the Centre for Economic Policy Research (CEPR) and the Department for International Development (DFID) [ERG #3155]; and
(iv) 2020/21 survey was funded by the Federal government of Nigeria through the FMSTI.

## CRediT authorship contribution statement

**Adedayo Olofinyehun:** Conceptualization, Data curation, Investigation, Methodology, Writing – original draft. **Abiodun Egbetokun:** Conceptualization, Investigation, Funding acquisition, Methodology, Data curation, Writing – review & editing. **Caleb Adelowo:** Investigation, Funding acquisition, Writing – review & editing.

## Declaration of Competing Interest

The authors declare that they have no known competing financial interests or personal relationships which have or could be perceived to have influenced the work reported in this article.

## Footnotes

1 By this we refer to the post-graduation performance of the alumni of these universities. Admittedly, these might not be the best among all universities in the country but they are the best available in our sample.

2 The compulsory entrepreneurship course policy only concerned universities.

3 https://www.gemconsortium.org/wiki/1599.

4 https://www.guesssurvey.org/datacollections/.

5 See https://beta.entrepreneurship.org.ph/2018/08/09/how-to-identify-your-personal-entrepreneurial-competencies/.
